# In vivo and In vitro evaluation of the antifungal activity of the PGPR *Bacillus amyloliquefaciens* RaSh1 (MZ945930) against *Alternaria alternata* with growth promotion influences on *Capsicum annuum* L. plants

**DOI:** 10.1186/s12934-023-02080-8

**Published:** 2023-04-13

**Authors:** Shereen A. Soliman, Reda E. Abdelhameed, Rabab A. Metwally

**Affiliations:** Botany and Microbiology Department, Faculty of Science, Zagazig University, Zagazig, 44519 Egypt

**Keywords:** Antioxidant defense, *Bacillus amyloliquefaciens* RaSh1, Electron microscopy, Fungicides, Leaf spot disease, Plant growth

## Abstract

*Alternaria alternata* that threatens pepper production and causes major economic harm is responsible for the leaf spot/blight disease. Chemical fungicides have been widely employed; unfortunately, fungicidal resistance is a current concern. Therefore, finding new environmentally friendly biocontrol agents is a future challenge. One of these friendly solutions is the use of bacterial endophytes that have been identified as a source of bioactive compounds. The current study investigates the in vivo and in vitro fungicidal potential of *Bacillus amyloliquefaciens* RaSh1 (MZ945930) against pathogenic *A. alternata*. In vitro*,* the results revealed that RaSh1 exhibited strong antagonistic activity against *A. alternata*. In addition to this, we inoculated pepper (*Capsicum annuum* L.) plants with *B. amyloliquefaciens* RaSh1 and infected them with *A. alternata*. As a result of *A. alternata* infection, which generated the highest leaf spot disease incidence (DI), the plant's growth indices and physio-biochemical characteristics significantly decreased, according to our findings. Our results also showed the abnormal and deformed cell structure using light and electron microscopy of *A. alternata*-infected leaves compared with other treatments. However, DI was greatly reduced with *B. amyloliquefaciens* RaSh1 application (40%) compared to pepper plants infected with *A. alternata* (80%), and this led to the largest increases in all identified physio-biochemical parameters, including the activity of the defense-related enzymes. Moreover, inoculation of pepper plants with *B. amyloliquefaciens* RaSh1 decreased electrolyte leakage by 19.53% and MDA content by 38.60% as compared to *A. alternata* infected ones. Our results show that the endophyte *B. amyloliquefaciens* RaSh1 has excellent potential as a biocontrol agent and positively affects pepper plant growth.

## Introduction

Plants are constantly threatened by a variety of pathogenic microorganisms present in their environments, such as bacteria, fungi, and viruses which cause diseases that significantly contribute to the overall loss in crop yield worldwide [1, 2]. Among these pathogenic microorganisms, fungi are the most dangerous biological stresses that cause harmful effects on crops quality and quantity leading to severe damage to agricultural crops in Egypt [3]. In response to fungal infection, there was a noticeable drop in plant physiological activity and chlorophyll concentration. In additions, this infection causes an imbalance in the movement of water and nutrients throughout the plant organs. As a result, this disrupts plant processes and leads to disease throughout the entire plant [4]. *Alternaria* is one of the most famous pathogens of fungal diseases causing a negative impact on vegetable crops [5]. *Alternaria* causes early blight and leads to a sharp decline in yield; the infection begins on the lower leaves of the plant first and then extends upwards, where small brown spots appear on the leaves [6, 7]. The most popular method of preventing fungal diseases is the application of chemical fungicides. However, these fungicides have several disadvantages, including high cost and potential impacts on the environment and human health [8, 9]. Additionally, the continued use of these pesticides has caused phytopathogens to become resistant [10, 11].

The use of microbial antagonists for biological control may offer a reliable, efficient, and environmentally friendly alternative method for the management of fungal diseases [12, 13]. Among these promising biocontrol agents are endophytes that have received increasing interest as the rhizosphere or phyllosphere acts as a source for numerous endophytes [14]. For a period of their life cycles, these endophytes live as symbionts in plant tissues asymptomatically [15]. Endophytes most likely use direct biocontrol strategies like antibiosis and competition as well as indirect strategies like inducing plant defense reactions against invasive diseases. Besides, they may stimulate plant development through phyto-stimulation and/or bio-fertilization [12, 16]. Endophytes are being evaluated for their capacity to produce new biologically active substances as well as their ability to maintain plant health due to their biological control and biological fertilizer qualities [16].

Additionally, endophytes have the ability to suppress disease, which may be introduced by the production of a variety of secondary metabolites, such as salicylic acid, siderophores, antibiotics, extracellular enzymes that break down the cell walls of pathogens, and the synthesis of volatile organic compounds, in addition to the induction of host plant-induced systemic resistance [17, 18]. Endophytes, according to Passari et al. [19], produce ammonia, phytohormones, hydrogen cyanide, siderophores, and solubilize phosphate, all of which are factors in encouraging plant growth.

Endophytic bacteria, including *Bacillus* sp., *Burkholderia* sp., *Enterobacter* sp., *Pseudomonas* sp., and *Serratia* sp., have been examined for their biocontrol potential against several plant diseases [20–22]. Kazerooni et al. [23] investigated *B. amyloliquefaciens'* capacity to promote *Capsicum* sp. growth and inhibit *Botrytis* grey mold and *Alternaria* leaf spot. Hazarika et al*.* [24] reported that *B. subtilis* SCB-1 screened for its antifungal potential against *Alternaria* sp., *Cochliobolus* sp., *Curvularia* sp., and *Fusarium* sp. Rashad et al. [16] observed that *B. amyloliquefaciens* GGA also had antagonistic action against *Sclerotium cepivorum* in vitro.

Due to their high nutritional value, peppers (*Capsicum annuum* L., family Solanaceae) are a well-known and significant economic vegetable crop [23, 25]. They are a rich source of vitamins, minerals, and antioxidants and assist to prevent inflammation, cancer, and cell damage. According to Attia et al. [26], fungi are one of the most dangerous pathogens of pepper plants. Pepper is vulnerable to a variety of fungal infections such as root rot, leaf spot, and wilt diseases [3, 27]; as a result, such stresses reduce yield and productivity, resulting in economic losses [28]. Previously, *B. amyloliquefaciens* RaSh1 (MZ945930) had been isolated from leaves of *Brassica oleracea* and it was reported for its ability to produce a number of bioactive compounds that have antifungal activities [10]. Consequently, the prime objective of our research is to assess antifungal capacity of *B. amyloliquefaciens* RaSh1 against *A. alternata *in vivo and in vitro as a biocontrol agent alternative to chemical products and further assess their growth-promoting effects on pepper plants in the greenhouse.

## Materials and methods

### Isolation, purification and morphological identification of the pathogenic *Alternaria* sp.

Leaves of diseased pepper plants (*C. annuum* L.) showing typical *Alternaria* leaf spot disease symptoms were collected in paper bags from several El-Sharkia governorate locations. Samples were surface sterilized for two min with 0.5% sodium hypochlorite solution before being cleaned with sterilized distilled water. After that, samples were dried between two sheets of sterile filter paper. Using a sterilized scalpel, the dried, sterilized spotted leaf tissues were chopped into small pieces along with the surrounding healthy tissues and put on the Potato Dextrose Agar (PDA) medium (Sigma-Aldrich, St. Louis, MO, USA) in 9 cm Petri dishes. Inoculated dishes were incubated at 25 °C for 4 days. Pure culture was obtained using a single spore culture technique according to Noman et al. [29]. The purified fungus was identified according to its morphological characteristics using the description of Ellis [30].

### Molecular identification of pathogenic *Alternaria* sp.

Using the Gene Jet Plant genomic DNA purification Kit, genomic DNA was isolated (Thermo procedure). Sigma Scientific Services Company (Cairo, Egypt) performed the PCR in a DNA Engine Thermal Cycler with a hot start at 94 °C for 3 min, followed by 30 cycles of 94 °C for 30 s, 55 °C for 30 s, and 72 °C for 60 s, followed by a final extension at 72 °C for 10 min. The sequencing was carried out by the GATC Company in Germany using an ABI 3730 × 1 DNA sequencer. The retrieved sequences were compared to the Gene Bank database using the NCBI BLAST programme. The 18S rRNA and ITS sequences in the Gene Bank database were compared to the sequences using BLASTN. The MEGA 6.0 programme was used to create a phylogenetic tree [31]. Finally, the sequence was uploaded to GenBank and assigned an accession number.

### Fungal inoculum preparation

*Alternaria alternata* inoculum was prepared from cultures grown on PDA medium then incubated at 25 °C for 7 days. The developed cultures were flooded using 50 mL sterilized water. The growth (mycelial mates and spores) was carefully scraped from the medium surface. The mycelium and obtained spore suspension were filtered through sterilized cheesecloth to eliminate mycelial fragments. The obtained fungal suspensions were adjusted to 10^5^ cfu/mL using the hemocytometer technique. Droplets of Tween 20 (0.5 mL/L) were finally added. Prepared suspension was used for spraying leaves in vivo.

### Bacterial inoculum preparation

*B. amyloliquefaciens* RaSh1 (MZ945930) was previously isolated from *B. oleracea* leaves*,* identified, and reported for its ability to produce a variety of bioactive compounds with antifungal properties [32]. *B. amyloliquefaciens* RaSh1 was cultivated in 1000 mL Erlenmeyer flasks with 250 mL of nutrient medium and incubated for 48 h at 37 °C on an incubator shaker at 100 rpm.

### Evaluation of the endophytic *B. amyloliquefaciens* RaSh1’s in vitro antagonistic activity

Using the dual culture plate method, *B. amyloliquefaciens* RaSh1’s antifungal activity was evaluated against *A. alternata*. Each PDA plate had a 6 mm-diameter disc of *Alternaria* sp. culture that had been grown for seven days in the center, with a loop of *B. amyloliquefaciens* RaSh1 streaking 1 cm from the plate's edge. Experimental controls included PDA plates that had only been infected with the fungal disc. The calculation of fungus growth inhibition using the following equation:

$${\mathbf{Growth}} \, {\mathbf{Inhibition}} \, \left( \% \right) \, = \, \left( {{\mathbf{R1}} \, {-}{\mathbf{R2}}} \right)/ \, {\mathbf{R1}} \times \, {\mathbf{100}}$$

*R1 represents the control plate’s inward linear growth, and R2 represents the dual culture plate's inward linear growth.

Antifungal assay of Thiram was done using poisoned food technique according to Mohammad et al. [33] with slight modification. 20 mL of sterilized melted PDA with 0.2% Thiram were poured in sterilized petri plates and agitated gently to become homogenized. The same procedure was done without Thiram as control. All petri dishes were allowed to solidify. Each Petri dish was inoculated with 10 mm mycelial disc of pure isolate taken from actively growing culture of *A. alternata*. Each mycelial disc was placed aseptically at the center of each petri plate. All Petri dishes were incubated at 25 °C for 5 days, at the end of incubation period the growth of mycelium was noticed for each treatment. Three replicates were maintained for each treatment.

### Pot experiment and leaves inoculation under greenhouse conditions

In a completely randomized design, the pot experiment was conducted in the greenhouse of the Botany and Microbiology Department, Faculty of Science, Zagazig University, with temperatures ranging from 23 to 30 °C and relative humidity from 60 to 85% with 10 replicates for each particular treatment. Five treatments (T1–T5) were conducted as described in Table 1. Plastic bags (15 × 25 cm) were filled with sterile field clay soil (2 kg/bag). On seedlings that were 40 days old, experimental treatments were used, and 1 seedling was transplanted per bag. *B. amyloliquefaciens* RaSh1 inoculum suspension was initially used to immerse the pepper seedlings’ roots for 4 h before to transplanting, and then bacterial inoculum (50 mL/bag) was used in irrigation for bacterial treatments. A fungal pathogen (*A. alternata*) was inoculated on the surface of healthy leaves two days after transplantation by pipetting individual droplets of a fungal suspension (10^5^ cfu/mL) onto the leaves. As a control, plants that were just sprayed and watered with tap water were employed. The infected plants were subjected to greenhouse conditions after being inoculated with the pathogen for 24 h to assure the infection process and maintain high humidity levels. The appearance of disease symptoms was noted four weeks following inoculation. Re-isolating *A. alternata* from diseased tissues and its identity was confirmed. The gathered samples were either used immediately or quickly frozen and preserved for further studies.

**Table 1:** Different treatments of the study are summarized as follows

Groups	Treatments
T1	Pepper treated with tap water (Control)
T2	Pepper infected with *A. alternata*
T3	Pepper infected with *A. alternata* and sprayed with Thiram (0.2%)
T4	Pepper treated with *B. amyloliquefaciens* RaSh1
T5	Pepper treated with *B. amyloliquefaciens* RaSh1 and infected with *A. alternata*

### Measurements

#### Assessment of disease incidence

The following formula was used to calculate the disease’s incidence (DI) according to Rashad et al. 16:

$$\mathrm{Disease\, Incidence }(\mathrm{DI}) \left(\mathrm{\%}\right)=\frac{\mathrm{Number \,of\, infected \,plants}}{\mathrm{Total \,number \,of \,plants}}\mathrm{ x }100$$

### Determination of morphological parameters

Pepper plants from the non-infected and *A. alternata* infected treatments were removed and rinsed with tap water 4 weeks after *B. amyloliquefaciens* RaSh1 application. We measured the pepper plant's shoot height and root length. The fresh weight (Fwt) was weighted and expressed by g. The samples were then heated to 70 °C for two days, during that time their dry weights (Dwt) were noted.

### Measurement of the physiological parameters

#### Estimation of chlorophyll content

The development of disease in pepper plants was also evaluated by examining the chlorophyll content (Chl a, Chl b, and carotenoids) by the Metzner et al. [34] protocol. Chopped-up pepper leaves (0.1 g) from healthy and infected plants were extracted with 85% acetone. Using a spectrophotometer, the absorbance at 644, 663, and 452.5 nm was measured to determine the amount of chl (WP 0803006). Then, using the Lichtenthaler and Wellburn [35] formulas, Chl a, b, and carotenoids were further determined.

$${\text{Chl}} a \left( {{\text{mg g}}^{{ - {1}}} {\text{leaf Fwt}}} \right) \, = \, \left[ {{12}.{7}\left( {{\text{OD663}}} \right) \, {-}{ 2}.{69 }\left( {{\text{OD644}}} \right)} \right] \, \times {\text{ V}}/{1}000 \, \times {\text{ W}}$$

$${\text{Chl}} b \left( {{\text{mg g}}^{{ - {1}}} {\text{leaf Fwt}}} \right) \, = \, \left[ {{22}.{9}\left( {{\text{OD644}}} \right) \, {-}{ 4}.{68 }\left( {{\text{OD663}}} \right)} \right] \, \times {\text{ V}}/{1}000 \, \times {\text{ W}}$$

$${\text{Carotenoids }} = \, \left( {{4}.{\text{2 OD452}}.{5}} \right) \, {-} \, \left( {0.0{\text{264 Chl}}.{\text{ a }} + \, 0.{\text{426 Chl}}.{\text{ b}}} \right) \, \times {\text{ V}}/ \, \left( {{1}000 \, \times {\text{ W}}} \right)$$

*OD refers to optical density, V to sample volume, and W to sample weight.

### Measurement of the water status of pepper plant leaves

The pepper leaves were collected and divided into small pieces (5–10 cm^2^), and each piece's Fwt was measured. They were then submerged in dist. water for around 4 h. Following that, the turgid weight of leaves (Twt) was determined individually. The leaves were then dried entirely for 72 h in an oven at 65 °C, and their Dwt was calculated. Measurements were made on the water content (WC), relative water content (RWC), and water saturation deficit (WSD) [36].

$$\mathrm{Relative \,Water \,Content }\left(\mathrm{RWC}\right)=\frac{(\mathrm{Fwt}-\mathrm{Dwt})}{(\mathrm{Twt}-\mathrm{Dwt})}\mathrm{ x }100$$

$$\mathrm{WC }(\mathrm{\%}) =\frac{(\mathrm{Fwt}-\mathrm{ Dwt}) }{\mathrm{ Fwt }}\mathrm{ X }100$$

$$\mathrm{WSD }\left(\mathrm{\%}\right)= 100-\mathrm{RWC}$$

### Leakage of electrolytes (EL) and the membrane stability index (MSI)

The method of Shi et al*.* [37] was used to estimate the plasma membrane permeability or electrolyte leakage (EL) of both diseased and non-diseased pepper leaves. To get rid of the electrolytes generated during leaf disc excision, ten leaf discs (10 mm in diameter) from young, completely expanded leaves were put in 50 mL glass vials and rinsed with distilled water. Then, 30 mL of distilled water was added to the vials, and they were left at room temperature for 24 h in the dark. At the end of the incubation time, the bathing solution's initial conductivity (EC1) was measured. Vials were heated at 95 °C in a water bath for 15 min, cooled to room temperature, and the EC2 was then calculated. Farooq and Azam [38] calculated the membrane stability index (MSI). The following formula was used to get the relative EL and MSI:

$$\mathrm{Electrolyte\, leakage }\left(\mathrm{EL}\right)=(E\mathrm{C}1/\mathrm{EC}2)\mathrm{ x }100$$

$$\mathrm{Membrane\, stability\, index }\left(\mathrm{MSI}\right)=[1-\left(\frac{E\mathrm{C}1}{\mathrm{EC}2}\right)]\mathrm{ x }100$$

*EC1 is a solution’s electrical conductivity before heating, and after heating, it is measured as EC2.

### Measurement of the biochemical parameters

#### Thiobarbituric acid reactive substances [TBARS] content

Using a thiobarbituric acid (TBA) reaction, the TBARS content, a marker of lipid peroxidation, was assessed in the pepper leaves of healthy and infected pepper plants [39]. A 0.6 mL of 0.1% (w/v) trichloroacetic acid (TCA) was used to homogenize 0.25 g of fresh leaf, and the mixture was then centrifuged for 15 min at 6000 rpm. The supernatant was heated at 95 °C for 30 min while being combined with 4 mL of 20% (w/v) TCA containing 0.5% (w/v) thiobarbituric acid (TBA), and then it was immediately transferred to a cold bath. Following a 10-min centrifugation of the extracted samples, the absorbance of the utilized supernatants was measured at 532 and 600 nm.

#### Assay of the defense-related enzymes activities

According to Qiu et al. [40], fresh pepper plant leaves (1 g) were mixed with 10 mL of an extraction buffer containing 100 mM potassium phosphate (pH 7.0), 0.1 mM EDTA, and 1% (w/v) polyvinyl pyrrolidone in order to assess the antioxidant enzyme activities in pepper plant leaves. The supernatant from centrifugation (12000 rpm for 15 min at 4 °C) was used to measure the enzyme activity. The consumption of H_2_O_2_ at 240 nm for two minutes was used to measure and assess the catalase (CAT; EC 1.11.1.6) activity [41]. A Lavid et al. technique [42] was used to find the activity of the enzyme polyphenol oxidase (PPO; EC.1.10.3.1). At 495 nm, the purpurogallin production was monitored.

#### 2,2-diphenyl-1-picrylhydrazyl (DPPH) radical scavenging assay

A known Fwt of leaf tissues (0.25 g) from healthy and diseased pepper samples was extracted with methanol and centrifuged for 10 min at 8000 rpm. The DPPH radical scavenging assay was used to determine the extracts' capacity to scavenge free radicals [43, 44]. A solution of 0.1 mM DPPH in methanol was prepared, and this solution was mixed with the methanolic extract at different concentrations (12.5–100 μg/mL). Methanol was utilized for the baseline correction, and the control was made in the same way as the above but without the sample extracts. After vigorous overtaxing, the reaction mixture was kept in the dark for 30 min. At 517 nm, the absorbance of the mixture was determined spectrophotometrically. Results were compared with ascorbic acid (ASA), a common antioxidant that was utilized as a reference. The following equation was used to compute the percentage of DPPH radical scavenging activity:

$$\mathrm{DPPH}\cdot \mathrm{scavenging \,effect }(\mathrm{\% of\, inhibition})=(A0-A1/\mathrm{A}0)\mathrm{ x }100$$

*The absorbance of the control is A_0_ and that of the sample extracts is A_1_.

Then, the percentage (%) of inhibition was plotted against concentration, and the IC50 was determined from the graph. The IC_50_ (the microgram of extract to scavenge 50% of the radicals) value was calculated using linear regression analysis.

#### Light and transmission electron microscopy (TEM)

Samples were collected from healthy (T1), infected (T2), and treated (T5) 1st and 3rd leaves and used for light and transmission electron microscopy examinations. The samples were first post-fixed in 2% osmium tetroxide for 2 h after being fixed in 2.5% glutaraldehyde in a 0.1 M sodium cacodylate buffer (pH 7) for 2 h [45]. The samples were then implanted in Epon-Spurr epoxy resin after being dehydrated in an escalating ethanol series and propylene oxide [46]. With the use of a Reichert Jung microtome, the semi-thin and ultra-thin sections were produced (Leica, Wetzlar, Germany). An analysis was performed using a compound microscope on the semi-thin sections. Uranyl acetate and lead citrate were used as the staining agents [47] for the ultra-thin sections and a Jeol 1010 TEM (Jeol, Tokyo, Japan) operating at 80 kV was used to investigate them.

### Statistical analysis

Results from 10 replicates (n = 10) are represented as means ± standard errors in the graphs. Analysis of variance (ANOVA) was used to statistically confirm the findings. Duncan's multiple range test (*p* < 0.05) was used to determine the significant difference between the control and treatment groups. With SPSS^®^ 18.0, the computations were carried out. Using SPSS, Pearson's correlation coefficients (*r*) were calculated to determine how growth indices and various biochemical markers interacted. Figures were assembled using OriginPro 8.5 for data analysis and graphing software.

### Results and discussion

The establishment of disease-controlling crop management techniques that are environmentally benign is a critical challenge. A biological alternative to antibiotics is the use of bacterial antagonists [48]. One of the most appropriate management techniques in the integrated management program is to lessen pesticide use in the environment [49]. PGPRs have been studied as plant disease biocontrol agents and, in addition, as stimulators of disease resistance in plants [50].

### Morphological characteristics of *A. alternata*

**Fig. 1:**
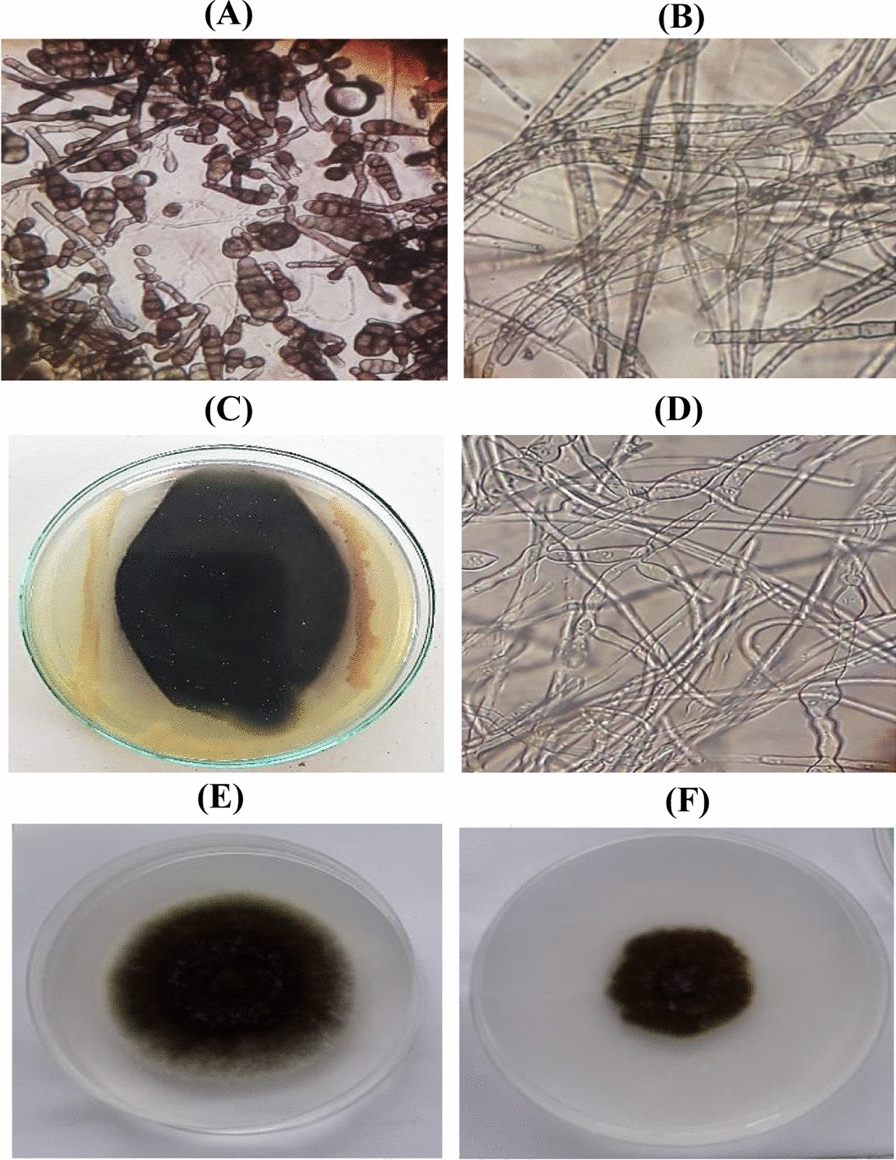
Morphological abnormalities in the mycelia of *Alternaria* sp upon interaction with *B. amyloliquefaciens* RaSh1 under the light microscope. Images **A** and **B** Untreated (control) *Alternaria* sp spores and mycelia. **C** Dual culture plate method showing inhibition of *Alternaria sp.* by *B. amyloliquefaciens* RaSh1. **D** Swelling and deformity of *Alternaria* sp mycelia treated with *B. amyloliquefaciens* RaSh1. **E** Untreated (control) *Alternaria* sp. cultivated on PDA media **F** *Alternaria* sp. cultivated on PDA media amended with 0.2% Thiram after 5 days of growth

The morphological traits of the pure culture of *Alternaria* sp. isolated from diseased pepper plant leaves were used to identify it. The purified culture of *Alternaria* sp. formed aerial mycelium that was greyish black in color with a black reverse. On microscopic examination, septate brown hyphae were seen, along with septate and brown conidiophores containing conidia in chains. Conidiophores were pale brown, simple, and branching, with catenulate conidia at the apex and fertile sections of the apex and apical fertile regions. Conidia were prosperous, acropetally formed, dark brown, cylindrical or spindle-shaped, often with cylindrical beaks, and muriform with 3–4 transverse walls and 1–2 longitudinal walls (Fig. 1A, B).

### Molecular identification of *Alternaria sp.*

**Fig. 2:**
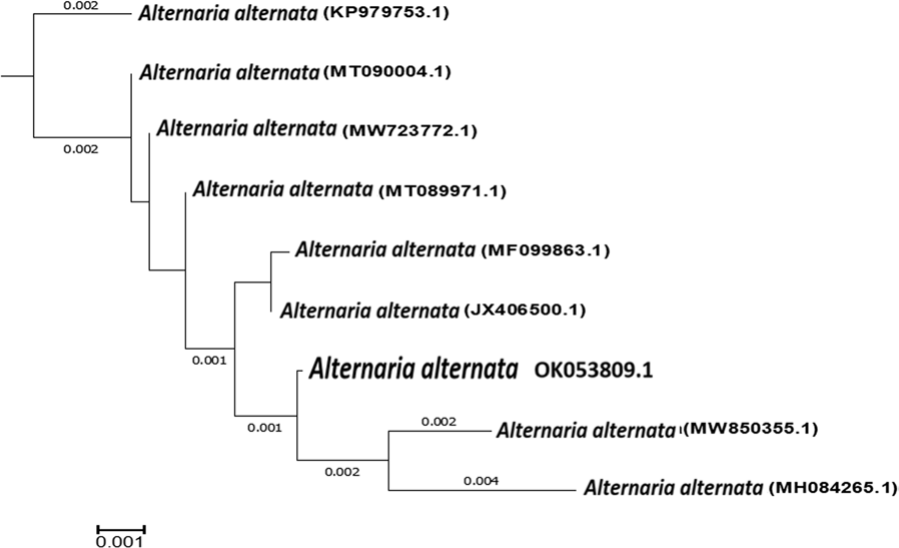
Phylogenetic tree of the 18S rRNA genes for *A. alternata* RaSh3 (OK053809.1) isolate and the others presented on GenBank based on the DNA sequence

The 18S rDNA gene sequence of an isolate of *Alternaria* sp. identified it as *A. alternata* RaSh3. As shown in Fig. 2, the acquired 18S rDNA gene partial sequence was entered into the GenBank database with the accession number OK053809.1.

### In vitro* antifungal assay*

The inhibition zone between *A. alternata* and *B. amyloliquefaciens* RaSh1was apparent in the dual culture test depicted in Fig. 1C. The obtained results indicated that *B. amyloliquefaciens* RaSh1 exhibited a strong antagonistic activity against *A. alternata* that proved its potent activity. Due to the release of antifungal metabolites, *B. amyloliquefaciens* RaSh1 has been examined for its fungitoxic action against a variety of soil-borne fungi [16, 32]. Among these metabolites that are produced by *B. amyloliquefaciens* are lipopeptides (e.g. bacillomycin, fengycin, and surfactin), hydrolytic enzymes, siderophores such as bacillibactin, and volatile compounds [8, 51, 52]. The feasible mechanisms of these metabolites’ actions include interference with cell membrane components, particularly sterol and phospholipid molecules, altering their structure and affecting membrane permeability [53]. Besides, inhibition of fungal DNA biosynthesis and cell lysis was also reported [16, 54].

Moreover, we observed a clear distortion and abnormality in the hyphal structure of *A. alternata* in the presence of *B. amyloliquefaciens* RaSh1 under light microscopy (Fig. 1D) as compared to the control one (Fig. 1B). Furthermore, as reported by Agarwal et al*.* [55] and Xu et al*.* [56], these outcomes suggest that antibiosis tends to be the biocontrol mechanism, employed by certain species of *Bacillus* against *F. oxysporum, A. alternata,* and *Helminthosporium sp.* Moreover, in this study, the antibiosis activity has been linked to the production of antifungal volatile compounds by *B. amyloliquefaciens* RaSh1 that are detected in its extract by GC–MS profiling, such as Bis (2-ethylhexyl) phthalate, Bis (2-ethylhexyl) ester, N,N-Dimethyldodecylamine, and Dibutyl phthalate [32]. These compounds are reported to have antimicrobial and antifungal activities against unicellular and filamentous fungi [57–59]. Antifungal assay of Thiram was done using poisoned food technique, results obtained in (Fig. 1 E, F) showed that Thiram has potent antifungal effect on the fungal linear growth.

### *B. amyloliquefaciens* RaSh1 ameliorative response to pepper during disease incidence (DI)

*A. alternata* causes some of the most devastating destruction to crops by destroying plants, reducing growth and causing diseases. They also produce mycotoxins that are detrimental to human health and livestock [60, 61]. *A. alternata* causes widespread leaf spot/blight, which has become a hazard to pepper production [62, 63]. The typical disease symptoms appear as small, brown spots on leaves. As the disease progresses, these spots may take on an irregular shape and gradually cover a large surface, causing the leaves to wither, dry out, and fall off [64].

**Fig. 3:**
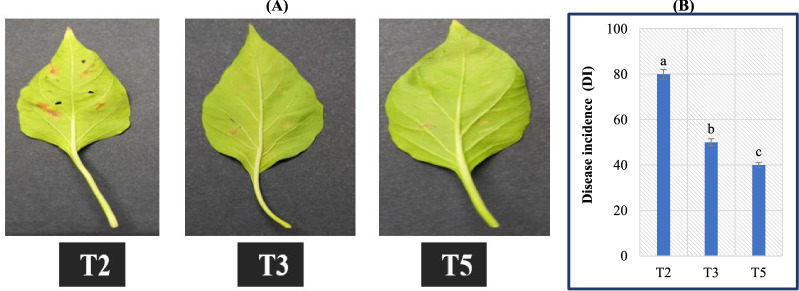
**A** Disease symptoms on pepper plant leaves infected with *A. alternata* (T2) and sprayed with Thiram (T3) or inoculated with *B. amyloliquefaciens* RaSh1 (T5). **B** Disease incidence percent (DI, %)

Synthetic chemical fungicides have been a mainstay in agriculture for controlling fungal diseases for decades; however, fungicides, like other pesticides, can have negative non-target effects on the environment, such as beneficial fungi to plant growth [65]. Wide fungicide use influences the mutualist fungi such as arbuscular mycorrhizal fungi [66, 67]. Our results showed that the inoculation of pepper plants with *B. amyloliquefaciens* RaSh1 bacterial endophyte significantly reduced the DI under *A. alternata* attack challenged conditions. Collected diseased samples were photographed and visible symptoms were also described as shown in Fig. 3(A), as the *A. alternata* strain was able to infect pepper plants, causing typical less spot symptoms. The highest DI (80%) was observed in *A. alternata* infected plants, while the lowest was recorded in plants infected with *A. alternata* and inoculated with *B. amyloliquefaciens* RaSh1 (40%) (Fig. 3B); the leaf spot disease symptoms were lessened by *B. amyloliquefaciens* RaSh1. *B. amyloliquefaciens* RaSh1 causes a great reduction in DI as compared to plants treated with *A. alternata* alone. Also, Thiram fungicide (0.2%) application decreased DI in *A. alternata* infected pepper plants (50%). However, in inoculated and non-inoculated *B. amyloliquefaciens* RaSh1 pepper plants grown under non-pathogenic conditions, certainly no disease symptoms were documented. Our finding was in agreement with previous reports which stated that endophytes are involved in controlling plant pathogens [22, 24, 68]. Additionally, Shahzad et al*.* [69] showed that *B. amyloliquefaciens* RWL-1 significantly reduced the growth of *F. oxysporum *in vitro when compared to the control.

### *B. amyloliquefaciens* RaSh1 ameliorative response to pepper growth attributes after *A. alternata* infection

The capacity of *B. amyloliquefaciens* RaSh1 to act as biocontrol agents was confirmed via a greenhouse experiment. *A. alternata* inoculated to pepper plants either in the presence or absence of *B. amyloliquefaciens* RaSh1. Besides, the effects of *B. amyloliquefaciens* RaSh1 application on pepper plant growth attributes are recorded in Table 2. Generally, all the assessed growth parameters (Fwt, Dwt of shoot and roots, shoot height, and root length) were significantly reduced in the pepper plants affected by leaf spot/blight, compared with the healthy control ones*.* Ghanbary et al*.* [70] reported that under optimal circumstances, plants dedicate their energy to growth, cellular preservation, and reproduction, but under pathogen invasion, they need to balance energy production with plant defense to ensure survival [71]. However, most of these parameters were significantly increased in plants bacterized with *B. amyloliquefaciens* RaSh1 compared with the control ones, regardless of whether the plants were infected with *A. alternata* or not. Similarly, there was a significant difference among the treatments in terms of root lengths and plant heights of the pepper plant. Unlike plant heights and Fwt, there was no significant difference in Dwt. In *A. alternata* infected peppers, application of *B. amyloliquefaciens* RaSh1 significantly improved shoot height (16.5 cm), shoot Fwt (3.99 g) and shoot Dwt (0.5258 g) as compared to infected one **(**Table 2**)**.

**Table 2:** Influence of *B. amyloliquefaciens* RaSh1 on growth promoting traits of pepper under *A. alternata* invasion.

Treatments	Shoot height (cm/plant)	Root length (cm/plant)	Fwt (g/plant)		Dwt (g/plant)	
			Shoot	Root	Shoot	Root
T1	17 ± 0.449^ab^	9 ± 0.238^b^	5.2 ± 0.137^a^	2.53 ± 0.067^b^	0.9616 ± 0.025^a^	0.3774 ± 0.0099^b^
T2	14 ± 0.37^d^	6.8 ± 0.179^d^	2.63 ± 0.069^d^	1.18 ± 0.031^d^	0.4941 ± 0.013^c^	0.1739 ± 0.0046^d^
T3	15.5 ± 0.41^c^	7.63 ± 0.202^c^	3.46 ± 0.092^c^	1.74 ± 0.046^c^	0.6393 ± 0.017^b^	0.2594 ± 0.0068^c^
T4	18 ± 0.476^a^	10 ± 0.264^a^	5.28 ± 0.139^a^	3.08 ± 0.081^a^	0.9749 ± 0.026^a^	0.455 ± 0.012^a^
T5	16.5 ± 0.436^bc^	8 ± 0.211^c^	3.99 ± 0.105^b^	1.89 ± 0.05^c^	0.5258 ± 0.014^c^	0.2766 ± 0.0073^c^

The values are the means of 10 replicates ± SE (n = 10). The same letter within each column indicates no significant difference between the treatments (*p* ≤ 0.05) as determined by Duncan’s multiple range test. Treatments: T1—control pepper (uninoculated), non-diseased; T2—infected with *A. alternata*, diseased; T3—infected with *A. alternata* and sprayed with Thiram (0.2%), diseased; T4—inoculated with *B. amyloliquefaciens* RaSh1), non-diseased; T5— inoculated with *B. amyloliquefaciens* RaSh1 and infected with *A. alternata*

The results of our pot experiment on the enhancing capacity of *B. amyloliquefaciens* RaSh1 for pepper plant growth are consistent with the findings of Rashad et al*.* [16], Shahzad et al*.* [69] and Zhang et al*.* [72] who found that *B. amyloliquefaciens* GGA, RWL-1 and IBFCBF-1 inoculation significantly enhanced all the growth traits of pepper, tomato and garlic plants under both diseased as well as non-diseased conditions of *Phytophthora* blight, *F. oxysporum* and *S. cepivorum* white rot*;* respectively. Also, in this regard, Kazerooni et al*.* [23] reported that *B. amyloliquefaciens* resulted in increased growth, improved plant health, and suppressed *Botrytis* gray mold and *Alternaria* leaf spot diseases produced by *B. pelargonii* and *A. alternata*. Therefore, *B. amyloliquefaciens* RaSh1 displayed strong antagonism toward *A. alternata* infection and improved the growth of infected pepper plants.

The extensively distinguished mechanisms of plant growth promotion caused by PGPR are phytohormone production, providing the essential nutrients, N_2_ fixation, and phosphate solubilization [19, 23]. Rangjaroen et al*.* [73] reported that *Klebsiella*, *Burkholderia,* and *Sphingomonas* endophytic bacteria enhance *Oryza* growth by increasing the production of indole-3-acetic acid (IAA) and gibberellins, phosphate solubilization and siderophore formation. Moreover, endophytic bacteria may promote plant growth through phytostimulation and/or bio-fertilization [12, 16]. Shahzad *et* *al* [69] and Srivastava *et* *al* [74] discovered that endophytes’ gibberellins, organic acid, and secondary metabolite producing capabilities provide additional support to plants and increase plant development, thus increasing their resilience to biotic and abiotic challenges. These mechanisms seem to contribute to the plant growth-promoting potential of *B. amyloliquefaciens* RaSh1 on pepper plants.

### Physiological parameters of pepper plants in response to *A. alternata *and *B. amyloliquefaciens* RaSh1

By taking over the plant’s physiology, endophytes can occasionally assist the host plant's defense mechanism against pathogenic microorganisms [75]. As the plant grows, it accumulates vigor and resilience to various abiotic and biotic challenges; this is one of the plant's defensive mechanisms against pathogens [76].

### Photosynthetic pigments in pepper plants

**Fig. 4:**
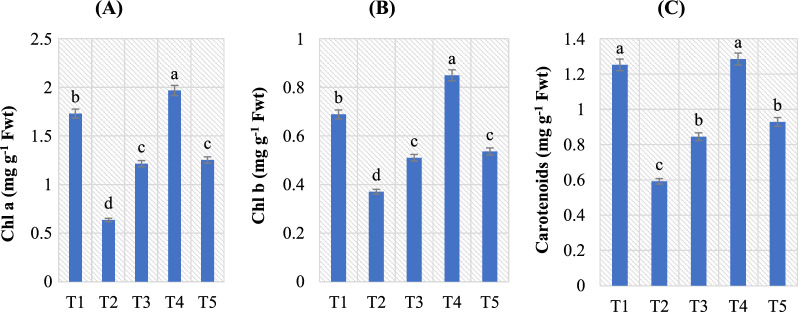
Effect of *B. amyloliquefaciens* RaSh1 and *A. alternata* on different pigment fractions; **A** Chl a, **B** Chl b and **C** Carotenoids of pepper plants. *The values are the means of 10 replicates ± SE (n = 10). The same letter above each column indicates no significant difference between the treatments (*p* ≤ 0.05) as determined by Duncan’s multiple range test. Treatments: **T1**—control pepper (uninoculated), non-diseased; **T2**— infected with *A. alternata*, diseased; **T3**—infected with *A. alternata* and sprayed with Thiram (0.2%), diseased; **T4**—inoculated with *B. amyloliquefaciens* RaSh1), non-diseased; **T5**—inoculated with *B. amyloliquefaciens* RaSh1 and infected with *A. alternata*

Leaf pigment content, which includes Chl and carotenoids, is a key measure of plant physiological status that could be used to assess photosynthetic activity [77–80]. Amounts of Chl a, Chl b, and carotenoids in pepper leaves in response to the different treatments are summarized in Fig. 4A–C. Infection of pepper plants with *A. alternata* led to decreases in the Chl content compared to untreated plants. The maximum reduction in Chl a content was observed in *A. alternata* infected pepper plants, followed by those *A. alternata* infected and treated with Thiram fungicide, according to the formulae suggested by Lichtenthaler and Wellburn [35]. The Chl b and carotenoids content followed the same pattern and were found to be maximally reduced (0.371 and 0.593 mg g^*−*1^ Fwt) in pathogen treated samples; respectively. These observed reductions are consistent with the findings of Hossain et al*.* [81] and Ghanbary et al*.* [82].

Reduced chloroplast numbers, chloroplast collapse, altered leaf photochemistry, and inhibition of Rubisco and other photosynthetic enzymes are all common biotic and abiotic stress responses [70, 82, 83]. These are usually because of photo-oxidation, Chl degradation, or impaired Chl biosynthesis [84], and our data suggest that these processes have occurred in pepper plant leaves as a result of *A. alternata* infection. Besides, reductions in stomatal conductance may also occur under *A. alternata* attack because of hyphal development blocking the stomatal opening [85]. As well, toxins generated by fungal infections like *A. alternata* can also impair the host's defensive response and limit leaf photosynthetic area [70].

However, these adverse effects on pigment synthesis in pepper plants were significantly mitigated by *B*. *amyloliquefaciens* RaSh1 inoculation. The Chl a and b quantities in the *B. amyloliquefaciens* RaSh1 treated pepper plants (1.254 and 0.537 mg g^*−*1^ Fwt) were greater than those of the non-*B. amyloliquefaciens* RaSh1 (0.636 and 0.371 mg g^*−*1^ Fwt) treated ones, whether the plants were pathogen-inoculated or not; so *B. amyloliquefaciens* RaSh1 application reduced the negative effects of the pathogen (*A. alternata*). Waqas et al*.* [86] showed that the Chl content of sunflower plant leaves was promoted in fungi-treated plants with or without the disease caused by *S. rolfsii*. Similar results were obtained by Shahzad et al*.* [69], who documented that *B. amyloliquefaciens* RWL-1 inoculation significantly improved the Chl content in tomato plants in comparison with *F. oxysporum*. Also, the inoculation of *B.* *subtilis* to diseased mung bean plants with *Macrophomina phaseolina* induced charcoal rot disease increased Chl content [87].

Moreover, Egamberdieva et al*.* [88] found that *B. subtilis* NUU4 inoculation causes 26% higher photosynthetic pigments in chickpea plant leaves compared to the un-inoculated ones. Photosynthetic pigments, particularly Chl, may be a good indicator of a plant's health and nitrogen status [89]. In previous studies, PGPR have been shown to boost the Chl content of plants by altering the amount of ethylene [90, 91]. Improved photosynthetic pigments in seedlings with endophytes may also be attributed to the plants' increased nutrient mobilization capabilities or by boosting the host nitrogen metabolism [91].

### Correlation between plant growth and photosynthetic pigments in pepper plants upon *A. alternata* and *B. amyloliquefaciens* RaSh1 applications

The Pearson correlation between Chl a, Chl b, shoot height and root length, shoot and root Fwt, and shoot and root Dwt revealed that *B amyliquefaciens* RaSh1 had a beneficial effect on pepper plants infected with *A. alternata* **(**Table 3**)**. Root Fwt (0.980), root length (0.966), shoot Fwt (0.967) and shoot height (0.920) showed significant positive correlations with Chl a. Chl b was found to have a significant positive correlation with root length, root Dwt, shoot Dwt and shoot length. Additionally, the correlation between shoot height, root length, and root Dwt was highly positive. Furthermore, there was a positive correlation between root length and root Dwt and shoot Dwt in plants.

**Table 3:** Pearson correlation coefficient (r) between some growth, physiological and biochemical parameters of pepper plants. Each square indicates the Pearson’s correlation coefficient of a pair of parameters

Parameters	Shoot height	Root length	Shoot Fwt	Root Fwt	Chl a	Chl b	RWC	WC	WSD	EL	MSI	PPO	MDA
Shoot height	1.000												
Root length	0.958^b^	1.000											
Shoot Fwt	0.926^b^	0.953^b^	1.000										
Root Fwt	0.921^b^	0.982^b^	0.969^b^	1.000									
Chl a	0.920^b^	0.966^b^	0.967^b^	0.980^b^	1.000								
Chl b	0.910^b^	0.985^b^	0.938^b^	0.986^b^	0.979^b^	1.000							
RWC	0.937^b^	0.827^b^	0.752^b^	0.740^b^	0.762^b^	0.754^b^	1.000						
WC	0.821^b^	0.731^b^	0.588^a^	0.632^a^	0.685^b^	0.692^b^	0.934^b^	1.000					
WSD	− 0.824^b^	− 0.803^b^	− 0.822^b^	− 0.834^b^	− 0.869^b^	− 0.836^b^	− 0.725^b^	− 0.660^a^	1.000				
EL	− 0.736^b^	− 0.789^b^	− 0.858^b^	− 0.876^b^	− 0.899^b^	− 0.847^b^	− 0.521^a^	− 0.445^a^	0.909^b^	1.000			
MSI	0.983^b^	0.943^b^	0.923^b^	0.920^b^	0.937^b^	0.905^b^	0.910^b^	0.822^b^	− 0.814^b^	− 0.774^b^	1.000		
PPO	0.264	0.095	− 0.051	0.030	0.081	0.084	0.490^a^	0.651^a^	− 0.387	− 0.087	0.263	1.000	
MDA	− 0.805^b^	− 0.819^b^	− 0.870^b^	− 0.874^b^	− 0.925^b^	− 0.866^b^	− 0.653^a^	− 0.599^a^	0.960^b^	0.974^b^	− 0.838^b^	− 0.225	1.000

^a^Correlation was significant at the* p* < 0.05

^b^Correlation was significant at the *p* < 0.01

### Effect of *B. amyloliquefaciens* RaSh1 on RWC in *A. alternata* infected pepper plants

The change in the water potential of leaves is a key indicator of a plant's water status, and it is clearly linked to the moisture content of the soil [92]. Pathogens that damage the vascular system of plants have an impact on it. In this study, we observed that infection of pepper plants with *A. alternata* resulted in a decrease in WC and RWC as compared to those grown under non-diseased conditions. Our findings are in line with those of Burman and Lodha [93], who showed that cowpea plants treated to concurrent drought and *Macrophomina phaseolina* infection experienced a notable decrease in the shoot water potential.

Considerably increased chlorotic leaf spots in the pathogen treatments reflect the strongly reduced WC and RWC, which refer to impaired cuticle integrity and increased non-stomatal water loss [70]. Increased water loss is also due to pathogen-secreted toxins inhibiting stomatal closure and decreased stomatal resistance, all of which diminish plant leaf water potential [94]. Our findings on electrolyte leakage provide additional support for this hypothesis (see below). RWC-based signs of severe water deficiencies in plants frequently lead to metabolic alterations such as photosynthetic impairment (as previously mentioned) (Table 3) and increases in respiration [95].

**Fig. 5:**
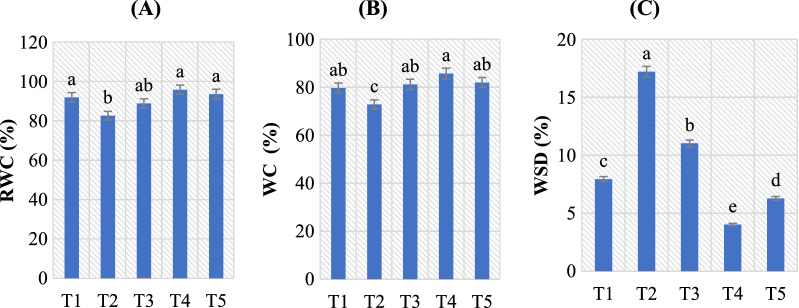
Effect of *B. amyloliquefaciens* RaSh1 and *A. alternata* on water status (%); **A** relative water content (RWC), **B** water content (WC) and **C** water saturation deficit (WSD) of pepper plant leaves under different treatments. *The values are the means of 10 replicates ± SE (n = 10). The same letter above each column indicates no significant difference between the treatments (*p* ≤ 0.05) as determined by Duncan’s multiple range test. Treatments: **T1**— control pepper (uninoculated), non-diseased; **T2**—infected with *A. alternata*, diseased; **T3**—infected with *A. alternata* and sprayed with Thiram (0.2%), diseased; **T4—**inoculated with *B. amyloliquefaciens* RaSh1), non-diseased; **T5**—inoculated with *B. amyloliquefaciens* RaSh1 and infected with *A. alternata*

Furthermore, *A. alternata* infection accelerated WSD in the diseased pepper leaves compared to control (Fig. 5). However, *B. amyloliquefaciens* RaSh1 inoculation decreased this parameter greatly. Also, we observed that *B. amyloliquefaciens* RaSh1 inoculated pepper plant leaves exhibited higher WC (85.87 and 82.10) and RWC (95.97 and 93.71) (*p* ≤ 0.05) under both control and *A. alternata* infection conditions (Fig. 5). This might be as a result of a decline in the pathogen’s inhibitory effect on pepper in the *B. amyloliquefaciens* RaSh1 inoculated plants. Our results are in accordance with Ghanbary et al*.* [70], who stated that the RWC of *Q. libani* seedling was significantly decreased by charcoal disease agents. According to Naveed et al. [20], the inoculation of *B. phytofirmans* with endophytic *Enterobacter* sp. greatly increased the RWC of maize under both normal and drought-stressed circumstances. Also, Dubey et al*.* [96] explored that there was a 35.3–48.41% rise in the leaf RWC in soybean seedlings inoculated with endophytes under drought stress conditions as compared to the control. These findings support investigations on PGPR-mediated reduction of osmotic stress [96].

### Effect of *B. amyloliquefaciens* RaSh1 on EL and MSI in *A. alternata* infected pepper plants

**Fig. 6:**
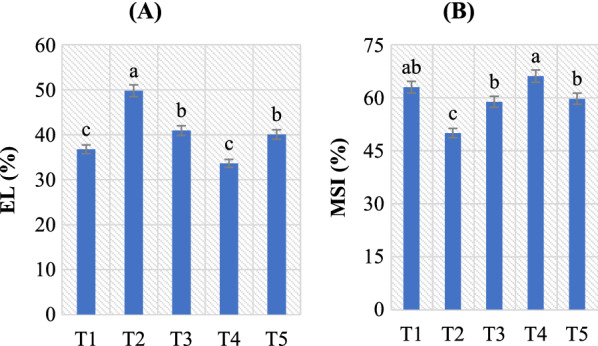
Effect of *B. amyloliquefaciens* RaSh1 and *A. alternata* on **A** electrolyte leakage (EL) and **B** membrane stability index (MSI) of pepper plant leaves under different treatments. *The values are the means of 10 replicates ± SE (n = 10). The same letter above each column indicates no significant difference between the treatments (*p* ≤ 0.05) as determined by Duncan’s multiple range test. Treatments: **T1**—control pepper (uninoculated), non-diseased; **T2**— infected with *A. alternata*, diseased; **T3**—infected with *A. alternata* and sprayed with Thiram (0.2%), diseased; **T4**—inoculated with *B. amyloliquefaciens* RaSh1), non-diseased; **T5**—inoculated with *B. amyloliquefaciens* RaSh1 and infected with *A. alternata*

The cuticle and stomata are physically disrupted by higher fungi and oomycetes, which also impede stomatal closing in the dark and are linked to an impaired stomatal opening in the light. This is because of the production of several toxins by these fungi that impair stomatal function [97]. Figure 6 shows that *B. amyloliquefaciens* RaSh1 inoculation decreased EL and its value ranged from 8.50 to 19.53% in pepper plant leaves under normal and diseased conditions, respectively, compared to control under normal and diseased conditions. Its maximum increase (35.42%) was observed after *A. alternata* infection in pepper compared to control, as *A. alternata* pathogen infection accelerated relative membrane permeability in the diseased pepper leaves. In this study, we found that both inoculated and un-inoculated plants' MSI decreased as a result of pathogen infection. However, *B. amyloliquefaciens* RaSh1inoculation significantly increased the MSI compared to the un-inoculated controls. According to Ghanbary et al. [70], *Q. libani* and *Q. infectoria* seedlings exposed to a combination of charcoal disease pathogens and drought showed the highest rise in EL. The first observable symptoms of pathogen infestation that cause disease are changes in membrane permeability and integrity [98].

**Fig. 7:**
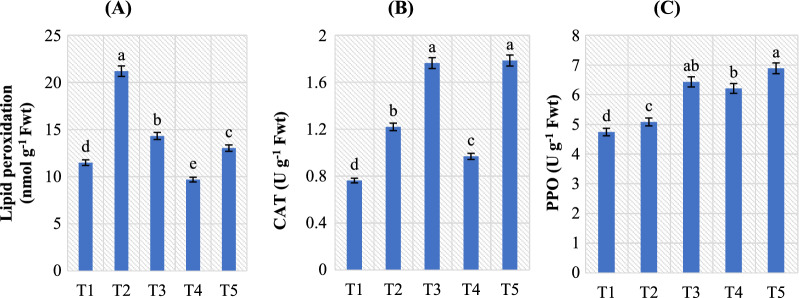
Effect of *B. amyloliquefaciens* RaSh1 on **A** lipid peroxidation (nmol/g Fwt) and the antioxidant activity of **B** CAT and **C** PPO in *A. alternata* infected pepper plants. *The values are the means of 10 replicates ± SE (n = 10). The same letter above each column indicates no significant difference between the treatments (*p* ≤ 0.05) as determined by Duncan’s multiple range test. Treatments: **T1**—control pepper (uninoculated), non-diseased; **T2**—infected with *A. alternata*, diseased; **T3**—infected with *A. alternata* and sprayed with Thiram (0.2%), diseased; **T4**—inoculated with *B. amyloliquefaciens* RaSh1), non-diseased; **T5**—inoculated with *B. amyloliquefaciens* RaSh1 and infected with *A. alternata*

Also, Naveed et al*.* [20] stated that *B. phytofirmans* besides *Enterobacter* sp. inoculation decreased the relative membrane permeability of maize under both normal and drought stress circumstances. Moreover, *B. amyloliquefaciens* RaSh1 inoculation resulted in an increase in MSI from 4.96% (without the pathogen) to 19.44% (with pathogen) compared to control or *A. alternata*-infected pepper plants, respectively (Fig. 6). In particular, *B. amyloliquefaciens* RaSh1 inoculation helped pepper leaves to maintain the relative membrane permeability besides reduced leaf damage compared to un-inoculated seedlings under *A. alternata* attack. Sandhya et al. [99] and Vardharajula et al. [100] found a positive association between stress sensitivity and membrane damage (EL). In addition, ROS that are produced during stress cause lipid peroxidation of membranes [20, 92, 101]. In our results, the *B. amyloliquefaciens* RaSh1 inoculation decreased the MDA content (as below) induced damage compared to control, fungicide application, and pathogen conditions (Fig. 7). Also, it is most feasible that RaSh1 inoculation colonization improved plant defense enzymes, to lessen the oxidative damage stimulated by *A. alternata* pathogen attack, suggesting that RaSh1 can enhance plant disease resistance. Similar to our results, Zhou et al*.* [102] found that *Pinus tabulaeformis* seedlings treated with PTD37 accumulated less MDA, broke down less Chl, and lost less tissue water than other treatments.

### Effect of *B. amyloliquefaciens* RaSh1 on lipid peroxidation in *A. alternata* infected pepper plants

The mobility of proteins, receptors, enzymes, and membrane ion channels is disturbed by the lipid peroxidation brought on by pathogenic stress [103]. As MDA is the marker for lipid peroxidation released from the cellular membranes of tissues and is formed by the reaction of ROS (H_2_O_2_ or/and O^−2^) with lipid molecules, the elevated concentration of ROS in the cellular system positively correlates with the oxidative changes affecting MDA content [92, 104–107]. According to our findings, *A. alternata*-induced leaf spot/blight resulted in a significant increase in the amount of lipid peroxidation produced in infected pepper plant leaves, followed by pepper plants infected with *A. alternata* and treated with Thiram fungicide and *B. amyloliquefaciens* RaSh1 treated samples over control plants **(**Fig. 7A**)**. Even though inoculation of pepper plants with *B. amyloliquefaciens* RaSh1 decreased MDA content in their leaves by 38.60% as compared to *A. alternata* infected ones; this reflects the positive interaction of *B. amyloliquefaciens* RaSh1 in pepper plants.

Therefore, *B. amyloliquefaciens* RaSh1 inoculation alleviated the oxidative stress in *A. alternata-*infected pepper plants. The biocontrol agents such as *B. amyloliquefaciens* RaSh1 can inhibit lipid peroxidation, which is what generates the oxidative burst caused by *A. alternata* in infected plants [87, 108]. Our findings support Lubaina and Murugan [109] finding that *A. sesame* infection increases the level of lipid peroxidation in the pathogen-inoculated leaf samples. Hashem et al*.* [87] reported that inoculation of *B. subtilis* to diseased mung bean plants with *M. phaseolina* inhibited MDA content while enhancing plant growth. These findings imply that the biochemical defense pathway is more in favor of the pathogen. The impact of *B. amyloliquefaciens* RaSh1 reduced the peroxidation of membrane lipids caused by *A. alternata* activation of excessive ROS generation in pepper plants. Thiram (2%) fungicide causes an increase in MDA content in diseased pepper plant leaves relative to control ones. This result was compatible with results of Metwally and Abdelhameed [67] in cucumber leaves treated with Ridomil. Thiram (2%) caused oxidative stress in pepper by producing ROS, which resulted in peroxidation of membranous lipids and the formation of MDA [110].

### Role of *B. amyloliquefaciens* RaSh1 and *A. alternata* on defense-related enzymes activities in pepper plants

When the antioxidant system fails to manage the intracellular ROS levels, oxidative stress and damage ensue; the generation of ROS as a result of this process can damage proteins and cell membranes, resulting in EL and ultimately cell death [103, 111–113]. The detoxification of ROS is necessary to safeguard critical cellular processes and the potential recovery of plants from oxidative damage [114]. The biocontrol strategy of *B. amyloliquefaciens* has also been shown to involve the induction of plant systemic resistance to invading pathogens. The effects of the applications of *B. amyloliquefaciens* RaSh1 on the activities of defense-related enzyme activities of pepper plants infected with leaf spot disease are summarized in Fig. 7. The antioxidant enzyme activities in pepper plants were significantly increased by the biotic interaction of *B. amyloliquefaciens* RaSh1 and *A. alternata*. The activity of CAT and PPO was considerably (Fig. 7B, C) increased to 60.31 and 7.14%, respectively, in *A. alternata-*infected pepper plants than in their control to scavenge the H_2_O_2_ produced due to pathogen attack.

In addition, further stimulation of CAT (46.55%) and PPO (35.65%) was observed in pepper plant leaves owing to *B. amyloliquefaciens* RaSh1 inoculation to mitigate the stress effects. Meanwhile, the greatest amounts of enzyme activities were for the *A. alternata* inoculated plants treated with *B. amyloliquefaciens* RaSh1, when compared with the control plants. The upregulation of antioxidant enzymes due to *B. amyloliquefaciens* RaSh1 inoculation protected the *A. alternata-*infected pepper plant’s metabolism by imparting fast removal of ROS. Our results are in accordance with Vellosillo *et* *al.* [115], who reported that pathogens such as fungi, bacteria, and viruses attack plants and produce hazardous ROS, which causes severe cell damage by triggering a sequence of destructive reactions.

Moreover, Li et al*.* [116] reported that cucurbit seedlings treated with *B. amyloliquefaciens* LJ02 reduced the *Sphaerotheca fuliginea* infection by triggering the defense-related enzymes biosynthesis. As a result, *B. amyloliquefaciens* RaSh1's effectiveness showed that pepper plants were more resistant to the leaf spot/blight disease because it prevented the initiation of the Haber–Weiss reaction, the formation of hydroxyl (OH^–^) radicals, and membrane dysfunction. Also, *B. amyloliquefaciens* RaSh1 can mediate the pepper plant’s growth by eliminating free radicals and maintaining photosynthetic rate, cellular redox potential, membrane integrity, and induced disease resistance in infected plants [108]. This suggests that the biocontrol activity of *B. amyloliquefaciens* RaSh1 against the pepper leaf spot pathogen was influenced by the induction of the host systemic resistance. It is apparent that the application of Thiram (2%) stimulates the antioxidant enzymes activities (PPO; 35.71%) in the diseased pepper plant leaves more than in control ones, as shown in Fig. 7, to mitigate the stress effects. According to Shakir et al*.* [117], the application of pesticides can quickly produce many types of physiological responses and oxidative damage in plants. It can also accelerate the formation of free radicals and ROS, which in turn causes oxidative damage [118].

### DPPH free radical scavenging activity

**Fig. 8:**
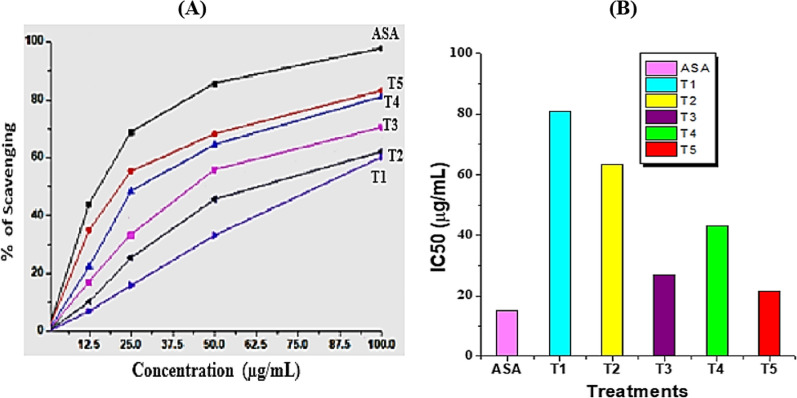
Effect of *B. amyloliquefaciens* RaSh1 and *A. alternata* on **A** DPPH radical scavenging activity and **B** IC50 of methanolic extracts of the different treatments: *ASA* Ascorbic acid; **T1**— control pepper (uninoculated), non-diseased; **T2**—infected with *A. alternata*, diseased; **T3**—infected with *A. alternata* and sprayed with Thiram (0.2%), diseased; **T4**—inoculated with *B. amyloliquefaciens* RaSh1), non-diseased; **T5**—inoculated with *B. amyloliquefaciens* RaSh1 and infected with *A. alternata*

Plants and pathogens both suffer significant oxidative damage as a result of plant-pathogen interactions that lead to a buildup of ROS in the cells. Both the host and the pathogen have created antioxidant systems to quench excessive ROS and keep ROS generation and scavenging mechanisms under control in order to repair this damage. Radical scavenging activities are very important to prevent the deleterious role of these radicals in different diseases. So, in our study, the DPPH free radical scavenging technique was used as an accepted mechanism for screening the antioxidant activity of methanolic extracts of pepper plants under different treatments (Fig. 8A, B). Figure 8A shows the free radical scavenging activity of the methanolic extracts of different treatments and standard ASA. *B. amyloliquefaciens* RaSh1 inoculated and infected with *A. alternata* pepper plant leaves possessed the highest free radical scavenging activity. At a concentration of 100 μg/mL, the scavenging activity in *A. alternata* infected peppers and treated either with *B. amyloliquefaciens* RaSh1 or Thiram (0.2%) was 83.19 and 81.08, respectively, compared to diseased (62.13%) or healthy (60.21%) ones (Fig. 8A).

The IC50 (half maximal inhibitory concentration) value was calculated to determine the concentration of the sample required to inhibit 50% of the radical [107]. The observed IC_50_ value of *A. alternata* infected plants and treated either with *B. amyloliquefaciens* RaSh1 or Thiram (0.2%) was 21.74 and 27.01 μg/mL, respectively, compared with *A. alternata* infected (63.45 μg/mL) and control (80.87 μg/mL) (Fig. 8B). The lower IC_50_ value indicates greater antioxidant activity as this value is inversely proportional to the free radical scavenging activity/antioxidant property of the sample. Our obtained results suggest that the combined treatment of *B. amyloliquefaciens* RaSh1 and *A. alternata* showed the highest radical scavenging activity (the lowest IC_50_), which indicates their electron transfer or hydrogen donating ability, and this was confirmed by the significant role of *B. amyloliquefaciens* RaSh1 in increasing the antioxidant enzymes (CAT and PPO) as previously mentioned in the results (Fig. 7). The effect of antioxidants on DPPH is thought to be due to their hydrogen donating ability, keeping in mind the role of polyphenols and tocopherols in scavenging the DPPH radicals by their hydrogen donating ability [119, 120].

### Correlation between biochemical and oxidative stress parameters

Inoculation of pepper plants with *B. amyloliquefaciens* RaSh1 had a positive effect on the plants subjected to *Alternaria* leaf spot disease conditions. This premise is supported by conducting a Pearson correlation analysis of the different biochemical and oxidative stress parameters that were measured **(**Table 3**)**. The results indicated that Chl a content in pepper plant leaves was positively correlated with MSI (0.937), RWC (0.762), WC (0.685), and Chl b content (0.979) and negatively correlated with MDA (-0.925), EL (-0.899) and WSD (-0.869). The Pearson correlation coefficients (r) were < 0.01 and < 0.05 for all of the compared attributes **(**Table 3**)**.

### Light and transmission electron microscopy (TEM)

**Fig. 9:**
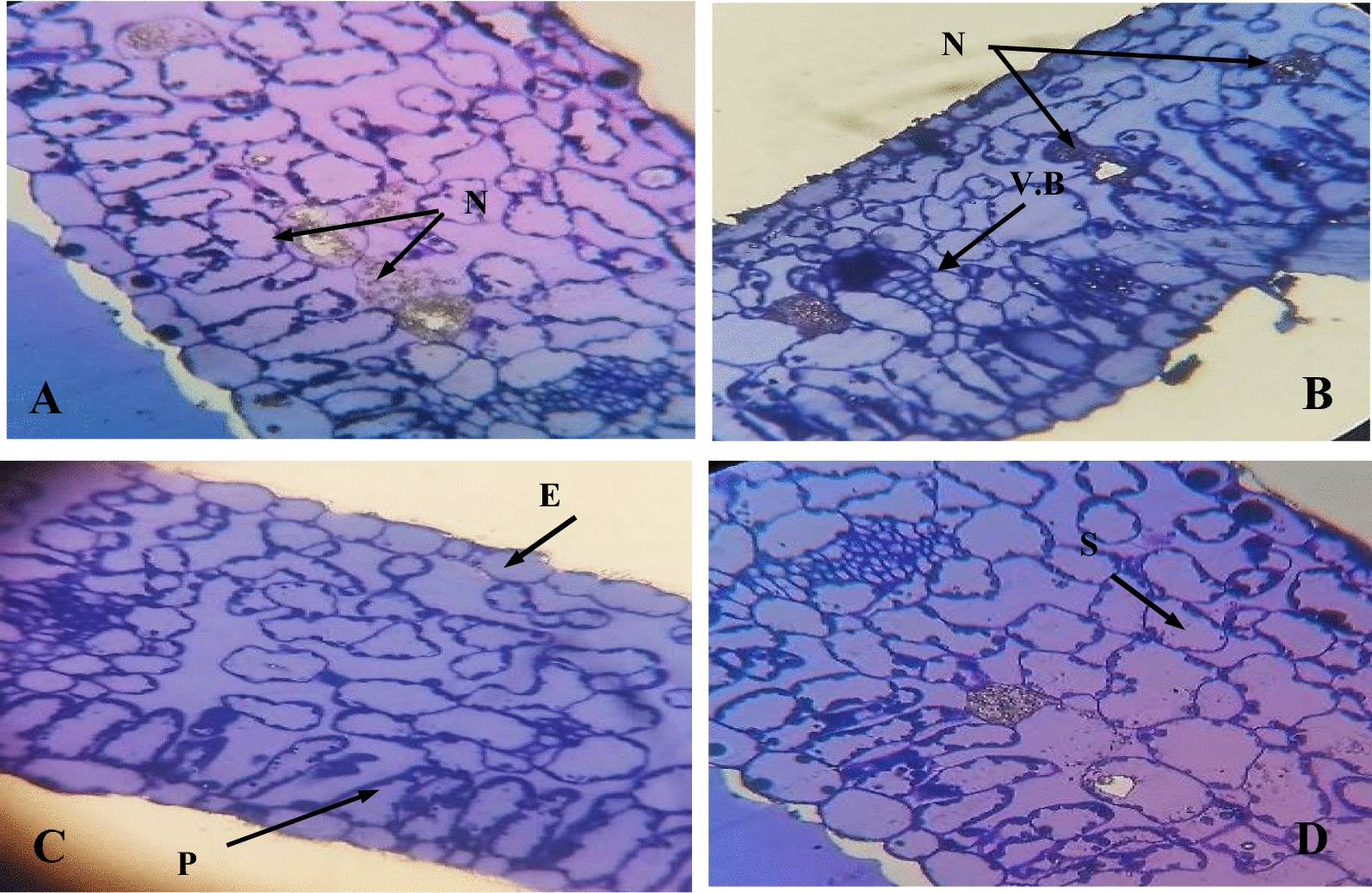
Light microscopy images showing the anatomy of pepper leaves **A** and **B** Necrotized inoculation sites of pepper leaves infected with *A. alternata* **C** Uninfected control leaves and **D** Pepper leaves infected with *A. alternata* treated with *B. amyloliquefaciens* RaSh1 (**MZ945930**) as biocontrol agent. **E**, epidermis; *N*, necrosis; *P*, palisade mesophyll; *S*, spongy mesophyll; *V.B*, vascular bundle

The movement of the pathogen toward the host, attachment to the plant’s surface, penetration of the host by the pathogen, and rapid proliferation of the pathogen once inside the host are necessary for a pathogen to successfully infect a host plant. Leaves collected from healthy pepper plants (T1), *A. alternata-*infected (T2), and those infected and inoculated with *B. amyloliquefaciens* RaSh1 (T5) (**MZ945930**) as biocontrol agent were examined at the structural and ultrastructural level using light and transmission electron microscopy. Structural changes of pepper leaf sections as detected by light microscope Fig. 9A–D) showed anatomical changes in the leaf tissues between different treatments.

**Fig. 10:**
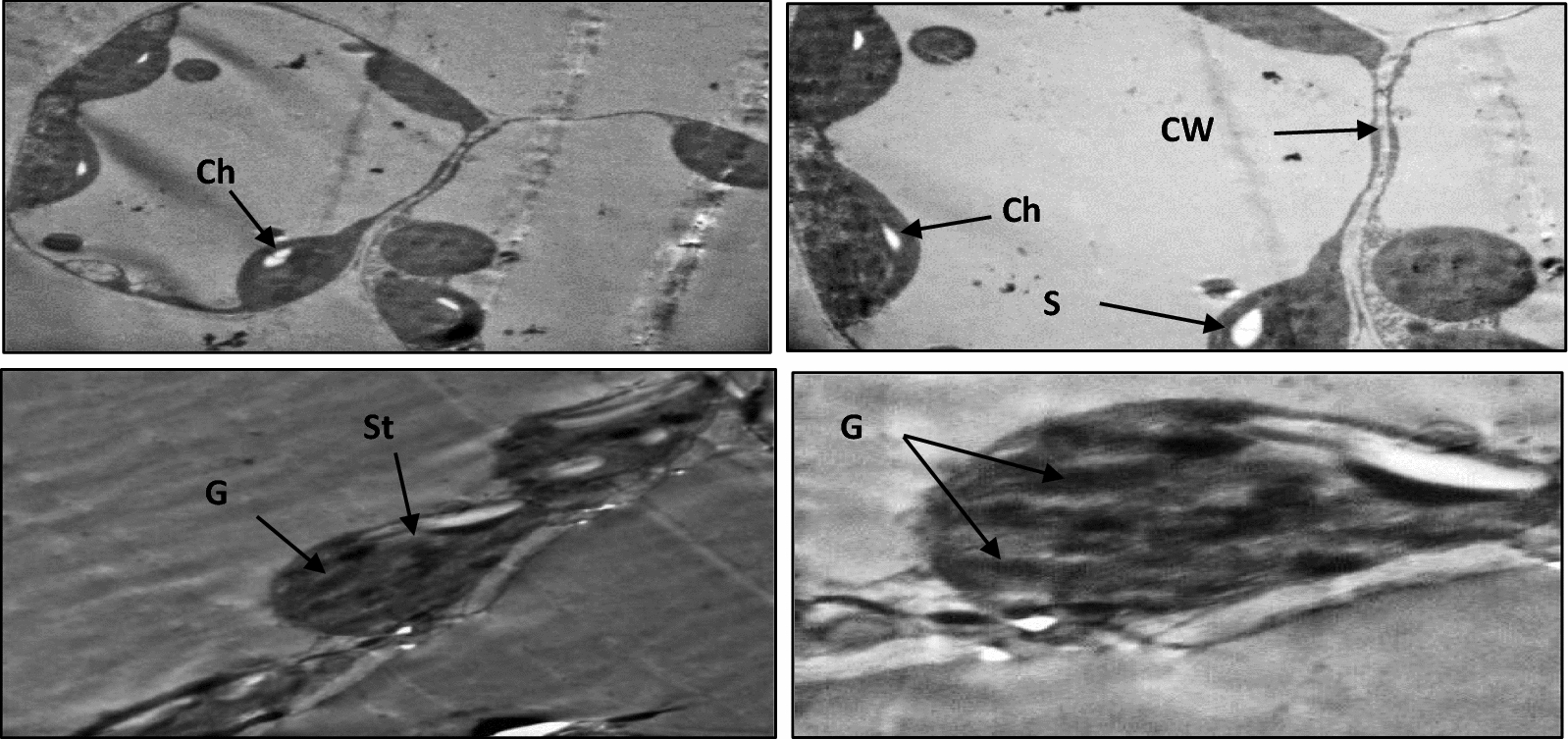
Transmission electron micrographs showing normal lenticular-shaped chloroplast with typical thylakoid and grana structures, and small starch grains in uninfected leaf (Control). *Ch* chloroplast, *CW* cell wall, *G* granum, *S* starch grain, *St* stroma

**Fig. 11:**
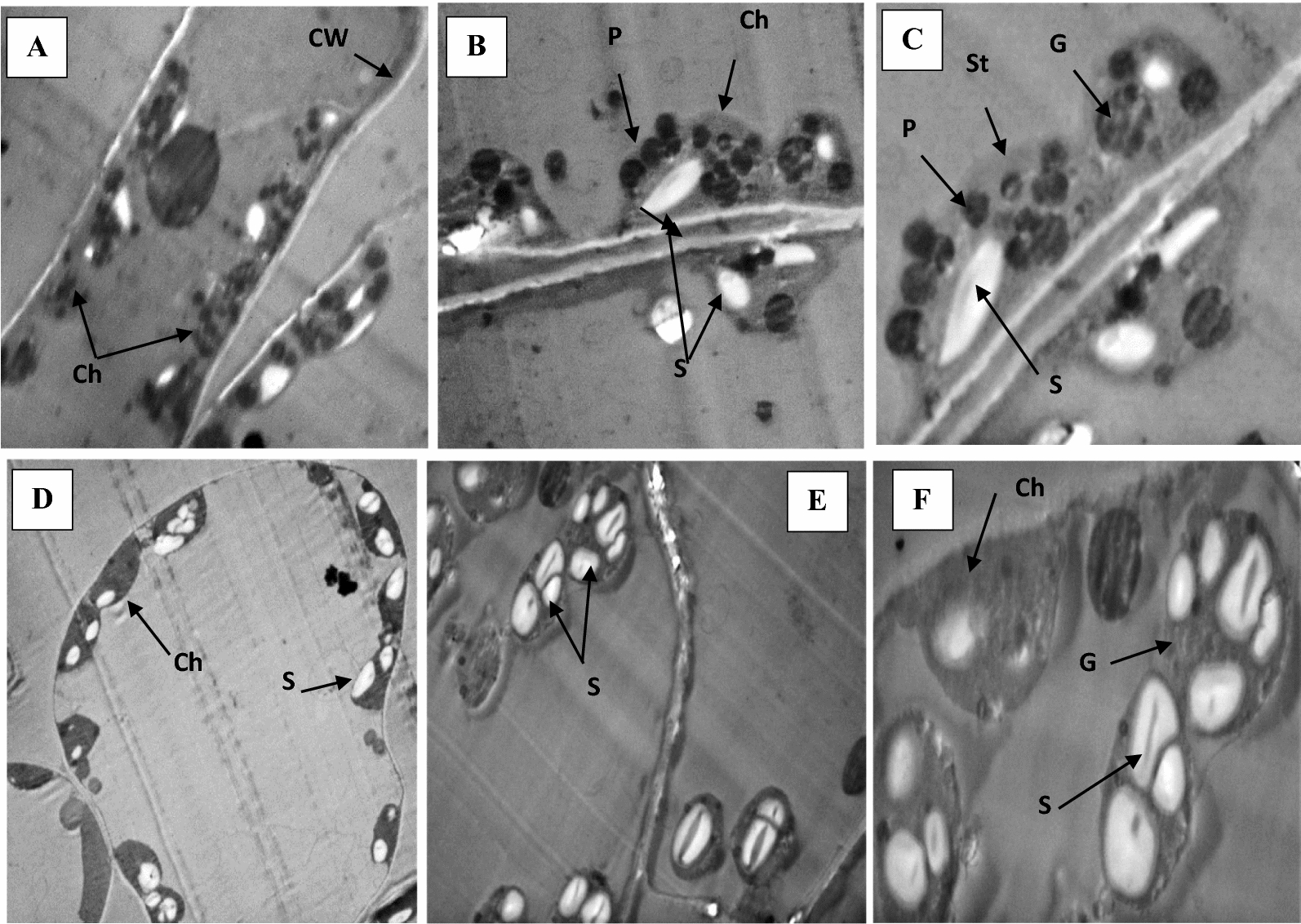
Transmission electron micrographs; **A**–**C** showing disorganization of chloroplast structure in the mesophyll cells of pepper leaves infected with *A. alternata* showing large starch grains and numerous plastoglobules, destroyed outer and inner membranes and collapsed grana and thylakoids. **D**–**F** showing chloroplast with preserved thylakoid and grana system and large starch grains in infected pepper leaves and inoculated with *B. amyloliquefaciens* RaSh1 (**MZ945930**) as biocontrol agent. *Ch* chloroplast, *CW* cell wall, *G* collapsed granum, P plastoglobule, *S* starch grain, *St* stroma

The ultrastructure of cells from healthy pepper leaves revealed that the mesophyll cells had a normal ultrastructure, with a large central vacuole and a narrow layer of cytoplasm, with normal classic chloroplasts characterized by a regularly arranged thylakoid, grana, well defined envelope, starch grains, and few plastoglobuli (Fig. 10). The same results have been previously described by Macioszek et al*.* [121]. Moreover, electron microscopic analyses of pepper leaves infected with *A. alternata* spores showed anatomical changes with severe damage in chloroplasts, abnormal enlarged plastoglobuli and starch grains and thylakoids degradation compared to the control leaves (Fig. 11A–C). However, chloroplast with preserved thylakoid, grana system and large starch grains were observed in leaves treated with *B. amyloliquefaciens* RaSh1(Fig. 11D, F). Similarly, disorganization of the membrane system of the chloroplasts in the infected mesophyll cells, breakdown of the envelope, damage of the cell wall, disappearance of starch grains or the appearance of large starch grains with an increase of plastoglobuli are indicative of infection [121–123]. Our results were also in agreement with Gabara et al*.* [124] and Kozieł et al*.* [125].

Successful infection of a susceptible host by a necrotrophic fungus depends on many environmental factors including host plant age and conidial concentration [126, 127]. Toxins and secondary metabolites may cause changes in the ultrastructure of organelles [128]. *A. alternata* pathotypes produce different HSTs (host-specific and non-host-specific toxins) which are responsible for the degradation of different organelles within an infected cell in over 200 plant species. Toxins can affect cell division, protein synthesis, membrane permeabilization and chloroplast photo-phosphorylation [129–131].

## Conclusion

Our results clearly demonstrate the significance of *B. amyloliquefaciens* RaSh1 for pepper development and performance, both in healthy and diseased conditions of *A. alternata* leaf spot/blight. This bacterial strain has the ability to release antioxidant enzymes and solubilize nutrients, which promote host development, physiology, and RWC while mitigating the disease in pepper plants. The *B. amyloliquefaciens* RaSh1 bacteria not only promotes pepper growth but also increases resistance to *A. alternata* infections. According to our findings, *B. amyloliquefaciens* RaSh1 application was effective and advantageous for *C. annum* under biotic stress (*Alternaria* leaf spot) and may be a candidate for the management of crop diseases. Given the increased disease tolerance against *A. alternata*, this suggests that the devastating effects of the leaf spot/blight disease caused by *A. alternata* in the *C. annum* are likely to get worse in the future.

## Data Availability

All datasets generated for this study are included in the manuscript. The relevant datasets supporting the results of this article are included within the article and the [GenBank NCBI] at https://www.ncbi.nlm.nih.gov/nuccore/OK053809.1

## Abbreviations

CAT: Catalase
DI: Disease incidence
Dwt: Dry weight
EL: Electrolyte leakage
Fwt: Fresh weight
MSI: Membrane stability index
PGPR: Plant growth promoting rhizobacteria
PPO: Polyphenol oxidase
WC: Water content
ROS: Reactive oxygen species
RWC: Relative water content
WSD: Water saturation deficit
